# Distribution of Relaxation Times Based on Lasso Regression: A Tool for High-Resolution Analysis of IMPS Data in Photoelectrochemical Systems

**DOI:** 10.1021/acs.jpcc.3c00770

**Published:** 2023-04-20

**Authors:** Alberto Piccioni, Pierpaolo Vecchi, Lorenzo Vecchi, Silvia Grandi, Stefano Caramori, Raffaello Mazzaro, Luca Pasquini

**Affiliations:** † Department of Physics and Astronomy, University of Bologna, Viale Berti Pichat 6/2, 40127 Bologna, Italy; ‡ Department of Mathematics, University of Bologna, Piazza di Porta San Donato 5, 40126 Bologna, Italy; § Department of Chemical, Pharmaceutical and Agricultural Sciences, University of Ferrara, Via Luigi Borsari 46, 44121 Ferrara, Italy

## Abstract

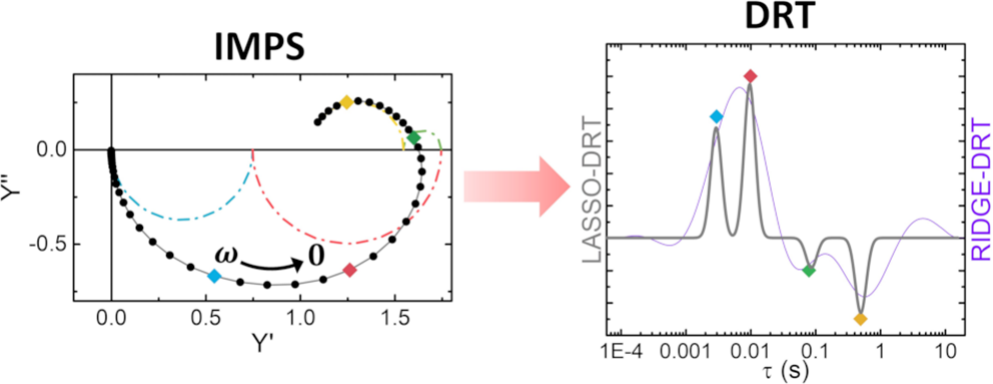

Intensity-modulated photocurrent spectroscopy (IMPS) has been largely employed in semiconductor characterization for solar energy conversion devices to probe the operando behavior with widely available facilities. However, the implementation of IMPS data analysis to complex structures, whether based on the physical rate constant model (RCM) or the assumption-free distribution of relaxation times (DRT), is generally limited to a semi-quantitative description of the charge carrier kinetics of the system. In this study, a new algorithm for the analysis of IMPS data is developed, providing unprecedented time resolution to the investigation of μs to s charge carrier dynamics in semiconductor-based systems used in photoelectrochemistry and photovoltaics. The algorithm, based on the previously developed DRT analysis, is herein modified with a Lasso regression method and available to the reader free of charge. A validation of this new algorithm is performed on a α-Fe_2_O_3_ photoanode for photoelectrochemical water splitting, identified as a standard platform in the field, highlighting multiple potential-dependent charge transfer paths, otherwise hidden in the conventional IMPS data analysis.

## 1 Introduction

Intensity-modulated photocurrent spectroscopy (IMPS) is a very powerful tool to assess the characteristic times that describe charge carrier dynamics of photoelectrochemical (PEC) cells and, more in general, photovoltaic devices. The theoretical basis of this technique applied to PEC devices was elaborated by Peter and co-workers, who published several articles^1−4^ between the 1980s and 1990s, where they developed a theoretical model based on kinetic equations whose solutions readily reproduce the IMPS spectra of a wide variety of cases, including systems involving single- and multistep processes. However, the application of a specific model to describe the system requires some a priori information, such as the expression of the reaction mechanisms, that may be missing. As a result, in most studies, a simplified analysis of IMPS data is mainly performed by adapting the system under investigation to the most trivial case, which consists in considering only two main kinetic processes, namely the hole transfer from the semiconductor’s surface to the electrolyte and the recombination of such holes with electrons coming from the semiconductor’s bulk.^5−7^ These processes are associated with two phenomenological rate constants, namely *k*^tr^ and *k*^rec^, embedding any available information about the kinetics of the underlying charge transport pathways. In the following, this simplified model will be called rate constant model (RCM), as previously introduced by Klotz et al.^8^ With this approach, any insight into multistep or parallel charge transfer paths is hindered. As this typically occurs in heterostructured photoelectrodes and heterojunctions, some information can only be extracted by selectively probing each layer of the junction, as recently suggested by our group.^9^

An IMPS experiment consists in measuring the frequency-dependent photocurrent *I*_ph_(ω) produced by the system in response to a small light perturbation, usually expressed as a photon flux ϕ_inc_(ω), and then calculating the response function:
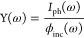
1

A DC light bias is also used to keep the system as close as possible to a real working condition. The amplitude of the sinusoidal light perturbation ϕ_inc_(ω) is usually kept below 10% of the intensity of the DC bias, in order to ensure the linear response of the photocurrent to the light stimulus. However, for most light conversion systems, the linear response of the photocurrent is, in general, not guaranteed when spanning within a wide light intensity range.^8,10,11^ Consequently, IMPS measurements can give significantly different results depending on the intensity of the DC light bias. When comparing characterizations and results from different experiments, it is therefore important to also consider the intensity of the DC light bias.

The standard analysis of an IMPS measurement is based on the graphical inspection of the shape of the IMPS spectrum in a Nyquist plot.^12^ Typically, the IMPS spectrum of a semiconductor/electrolyte interface shows two semicircles: one in the fourth quadrant at high frequencies and the other in the first quadrant at lower frequencies. According to the RCM, this last semicircle contains fundamental information about the surface kinetics of the system: the frequency ω_max_ that corresponds to the maximum of the semicircle is
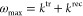
2meanwhile the intercept at medium frequencies (MFI) with the *x*-axis imaginary part of IMPS curve = 0) represents the product of the light-harvesting efficiency (LHE) multiplied by the charge separation efficiency (CSE). The intercept at lower frequencies (LFI) represents the external quantum efficiency (EQE), which, when divided by the MFI,gives the transfer efficiency:^13^
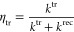
3

Consequently, this analysis relies on the presence of a maximum and intercept values of the IMPS spectrum, even though the extraction of these values is not always straightforward.

A potentially more effective method for the analysis of IMPS data is the distribution of relaxation times (DRT) algorithm, which is usually adopted for analyzing electrochemical impedance spectroscopy (EIS) measurements.^7^ This method enables an assumptions-free understanding of the frequency-domain response function, allowing for a quantitative analysis of both simple and complex systems. The DRT algorithm for fitting EIS data is usually adopted in systems like fuel cells^14,15^ and batteries^16−18^ to extract their characteristic times. The standard DRT algorithm is based on ridge regression, which ensures a good fit of EIS spectra, avoiding overfitting and oscillations of the DRT curve.^19^ An important boundary condition that comes from the physics of these systems is that the values of the DRT curve fall in the positive domain of real numbers. The physical meaning of this restriction is that the response function measured with EIS is modeled as a true electrical impedance *Z*(ω) = *V*(ω)/*I*(ω) of an equivalent circuit composed only by resistors and capacitors, and in this case, only positive values of real impedance and negative values of imaginary impedance are allowed. There are only a few cases where the equivalent circuit can also include inductors, such as in polymer electrolyte membranes, that show a typical circle in the fourth quadrant at low frequency.^20^ However, in IMPS measurements usually performed on PEC systems, the measured response function has the form of an admittance, as reported in eq 1. In this case, both positive and negative values for the real^21^ and imaginary^2^ component of *Y*(ω) are possible. The use of standard DRT analysis based on ridge regression to fit *Y*(ω) is not new in literature^8,10^ but this results in an over-oscillating DRT curve when the domain is extended to negative values of the DRT. This problem is well known, and it is intrinsic in the use of the ridge penalty term in the minimization problem. A common strategy to overcome this problem is dividing the IMPS spectrum into two semicircles, one in the positive and the other in the negative quadrant of the Nyquist plot and performing the DRT separately on single semicircles.^7,8^

In this article, we propose a method to improve the analysis of IMPS using a modified DRT algorithm based on Lasso regression. The specific features of this type of regression applied to DRT analysis of EIS data have been already explored by Ciucci et al.^19^ While ridge regression was assessed to ensure a smooth and well-fitting DRT curve even in the case of noisy data, avoiding overfitting, Lasso regression was observed to return a discrete DRT time spectrum, suitable for selecting only a few characteristic times of the system. Hereby, we demonstrate how such peculiar features can be applied to IMPS spectra analysis, where the main interest consists in extracting only a few rate constants from our systems. Furthermore, the issue of an over-oscillating DRT curve resulting from ridge regression is essentially bypassed, producing reliable analysis in presence of negative values for *Y*(ω).

## 2 Results and Discussion

A detailed mathematical description of the proposed DRT algorithm based on Lasso regression (L-DRT) is reported in the Supporting Information (SI). The algorithm was implemented in Python and it is available here.^22^ In the following, we show how the application of this algorithm can greatly improve the analysis of the response function of three different systems: (a) a simulated discrete system, made of a specified number of characteristic times, (b) a generalized physical RCM^1^ described by kinetic equations and (c) a real case of hematite photoanodes used for water splitting in a PEC cell.

### 2.1 Simulated Discrete System

The first benchmark to validate the algorithm is a simulated dummy system made of *N* different characteristic times, which is equivalent to an electrical circuit consisting of a series of *N* Voigt elements (i.e., parallel RCs). Every Voigt element is characterized by a characteristic time τ_*n*_ = *R_n_C_n_* and a weight, given by the value *R_n_* of the resistance. The impedance of such a circuit is:
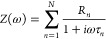
4

Equation 4 is equivalent to eq S2, upon substituting *R_n_* with *g*(τ_*n*_). The main difference is that *g*(τ_*n*_) can also be negative in the IMPS case. Therefore, IMPS data was generated using eq S2 with *N* = 4. In Table 1, the values used for the calculation, are reported.

**Table 1:** Values of τ_*n*_ and *g*(τ_*n*_) Used for Simulating the IMPS Spectrum^a^

*n*	τ_*n*_	*g*(τ_*n*_)	peak height (Lasso)
1	3 ms	0.75	0.73
2	10 ms	1	1.01
3	80 ms	–0.2	–0.19
4	500 ms	–0.5	–0.49

a In the last column, the height of the peak obtained using the L-DRT algorithm is reported.

**Figure 1:**
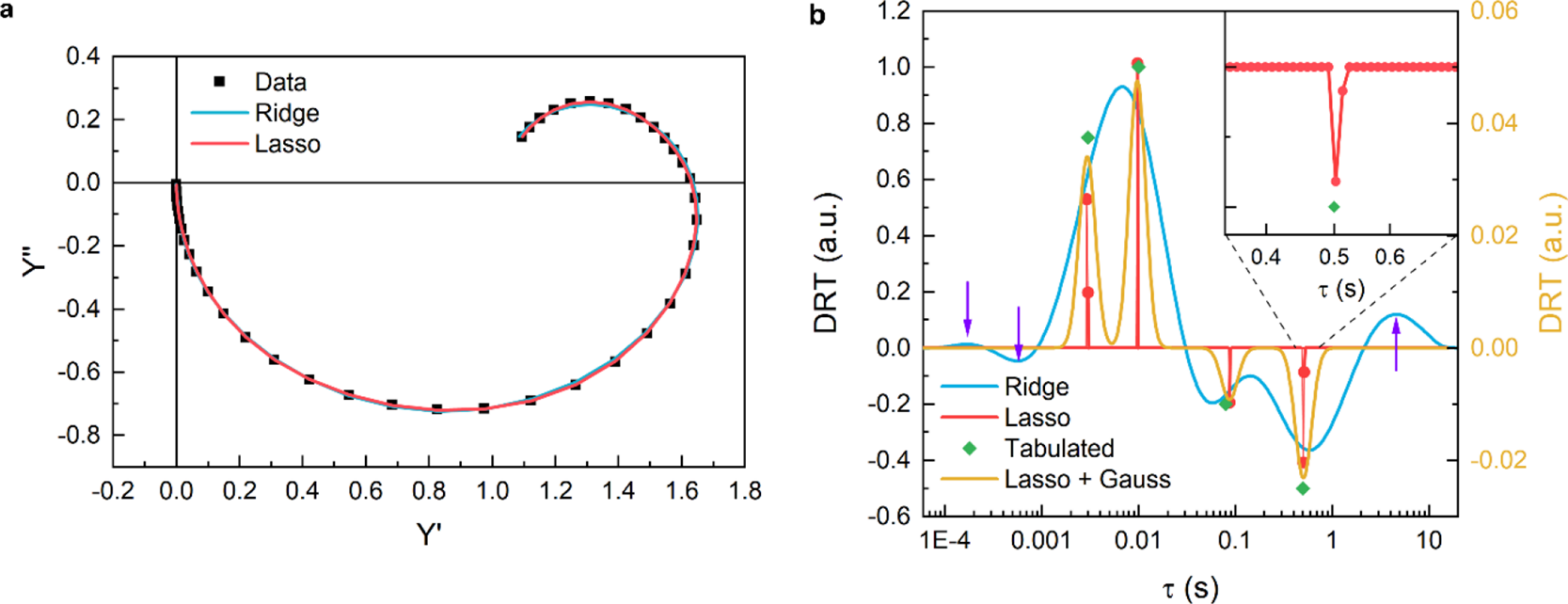
(a) IMPS spectrum generated using eq S2 and values in Table 1, overlapped with fit curves obtained using Lasso and ridge regression, (b) DRT curves calculated using Lasso and ridge regression, together with the Gaussian convolution of the L-DRT (GL-DRT curve); tabulated values (τ, *g*(τ)) used to generate the IMPS spectrum are reported as green points. Notice that the *y*-scale of the GL-DRT curve (yellow) differs from the one of the discrete L-DRT curve (red) since the peak of every Gaussian centered at τ is normalized so that its integral is equal to the value *g*(τ).

The IMPS spectrum generated using eq S2 and shown in Figure 1a displays two semicircles, one in the negative and one in the positive imaginary admittance quadrant, suggesting a system defined by two characteristic times. However, the characteristic times used to simulate this spectrum are four, two negatives and two positives, clearly pointing out how a simple graphical evaluation of such an IMPS spectrum is not reliable for identifying the number of elements characterizing the circuit.

By applying DRT analysis and fitting this spectrum with Lasso and ridge regression, a good reconstruction of the data is obtained, as shown in Figure 1a. In fact, the blue and red lines fully match every point of the IMPS spectrum.

By focusing on the DRT curves, a huge shape difference between the curves calculated with the two methods (Figure 1b) is clearly visible. On one hand, ridge regression is not able to deconvolve the first two positive peaks. On the other hand, even if the two negative peaks are actually deconvolved, their positions are shifted with respect to τ_3_ and τ_4_ due to the oscillations that give rise to other peaks. In addition, without prior information on the system, the oscillations introduced by ridge regression (see arrows in Figure 1b) may be misinterpreted as real elements of the analyzed circuit, introducing an artifact. On the opposite, Lasso regression is able to find correctly not only the four main peaks centered exactly at τ = τ_*n*_, but also to return their precise height. In fact, as reported in Table 1, there is a good agreement between the tabulated values *g*(τ_*n*_) and the height of the peak given by the L-DRT algorithm. However, it must be noticed that the peak centered at τ_1_=3 ms and τ_4_=500 ms are split into two close points (see inset Figure 1b). For the sake of clarity, when a peak is split into two or more points, we report the sum of their height. In order to have a more reliable reconstruction of the data and avoiding the multiplication of peaks (i.e., overfitting), it is useful to build on each point τ of the L-DRT a Gaussian curve centered on τ and with FWHM, which is equal to the logarithmic spacing between two consecutive frequencies used for the measurement, namely FWHM = *S* × ( log τ_*n* + 1_ – log τ_*n* – 1_), where *S* is a parameter introduced in the SI. The height of the Gaussian is then normalized so that its integral is equal to the value *g*(τ). Therefore, the resulting DRT curve will be the sum of several Gaussian curves; most of them with a height close to zero, and only few of them, with a height appreciably different from zero (those centered at τ_*n*_). In the following, we will refer to this curve as the Gaussian Lasso DRT curve (GL-DRT curve). This representation bestows a more reliable description of the simulated dummy system, pointing out the superior solidity of the Lasso approach with respect to conventional ridge regression.

### 2.2 Generalization of the Rate Constant Model

From the previous application, we saw that DRT analysis based on Lasso regression is capable of deconvolving close characteristic times and returning the right intensity of the relative process. These peculiar features are now exploited to analyze IMPS spectra calculated using a generalization of the RCM proposed by Peter,^1^ which describes the photocurrent response of a semiconductor/electrolyte interface to a periodic illumination of the photoelectrode. In the RCM, all the minority charge carriers generated after the light excitation are supposed to accumulate homogeneously along the surface of the semiconductor, then either undergo a charge transfer process to the electrolyte with a rate constant *k*^tr^, or recombine with majority carriers coming from the bulk with a rate constant *k*^rec^. However, in a more realistic picture of the system, a semiconductor’s surface is not homogeneous, causing a distribution of accumulation sites for minority carriers. Examples of such systems are very common since they are the results of the nanostructured surfaces, decoration with catalysts, or heterojunction with porous layers.^6,23,24^ Each accumulation site *n* is therefore characterized by a fraction *p_n_* of total hole flux *I*_hole_ toward the surface, so that , and two rate constants *k*_*n*_^tr^ and *k*_*n*_^rec^. No charge redistribution among different accumulation sites is considered during the experiment. This assumption may be valid only when dealing with a limited number of independent surface sites, while more complex models may be required for continuous distribution of interdependent sites.

**Figure 2:**
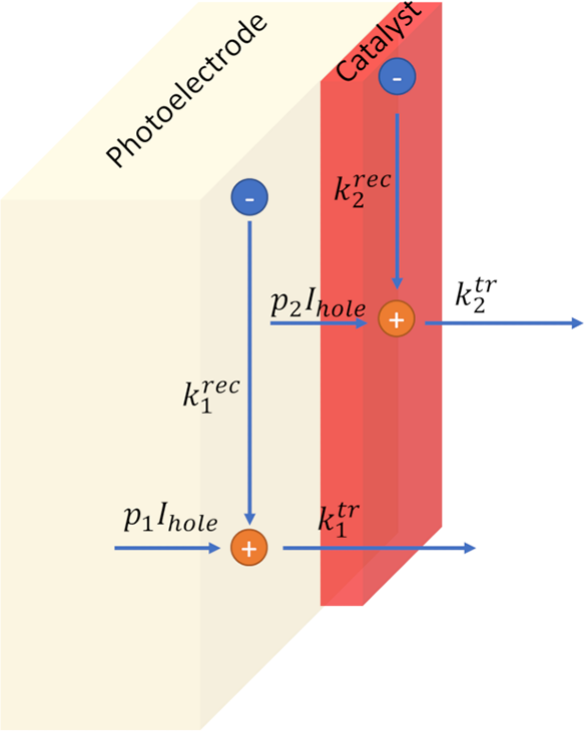
Model of the dynamic processes of charge transfer and recombination when two hole accumulation sites are present. In this case, holes are supposed to accumulate and transfer both directly from the surface of the photoelectrode (*p*_1_) or from the catalyst, which account for an additional hole transfer/recombination pathway (*p*_2_).

**Figure 3:**
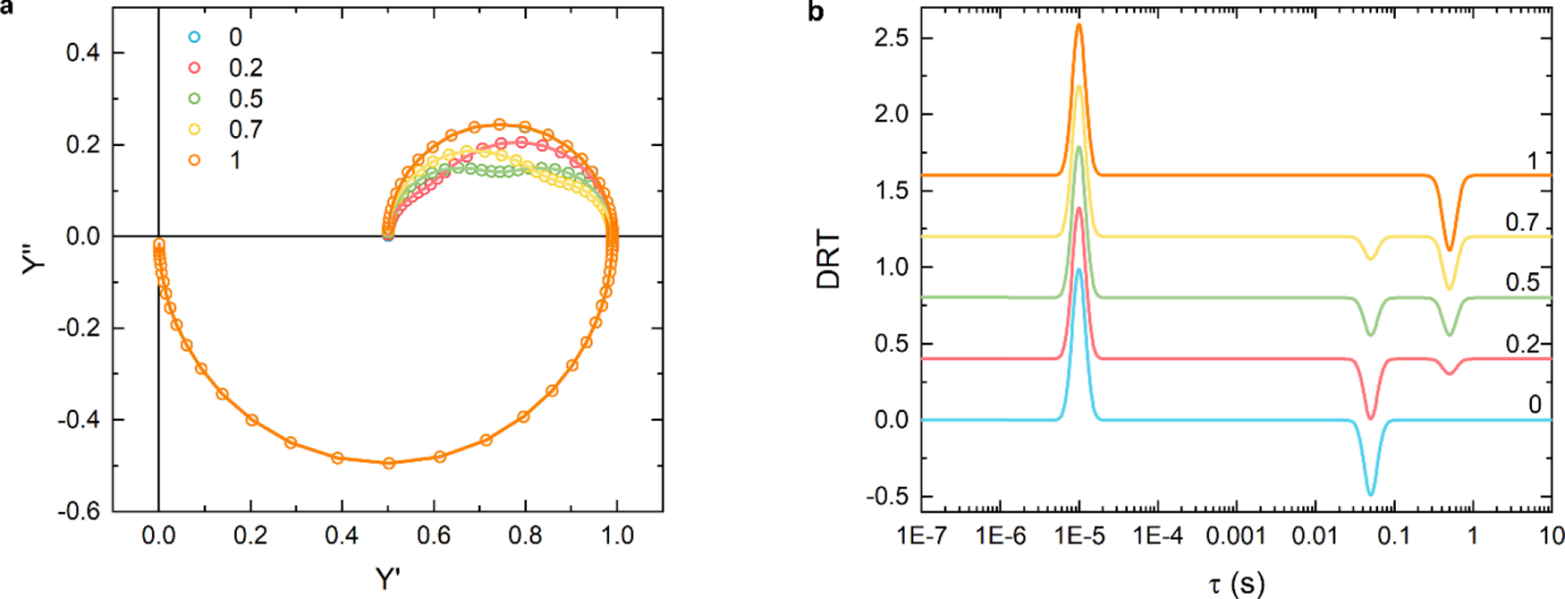
(a) IMPS spectra generated using τ_cell_ = 10^–5^ s, *p*_1_ = 0,0.2,0.5,0.7, and 1 and *N* = 2 with *k*_1_^tr^ = *k*_1_^rec^ = 1 and *k*_2_^tr^ = *k*_2_^rec^ = 10, together with their fit curves obtained using λ = 0.5. (b) GL-DRT curves (in this case positive peaks are normalized to 1).

The general equation that describes the frequency-dependent part of the total photocurrent is
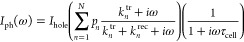
5where τ_cell_ = *RC* represents the characteristic time of the cell and it is determined by the electrode capacitance *C* and the total series resistance *R* associated with the electrolyte and ohmic contact. If *N* = 1, eq 5 returns the simple RCM, where a single state is responsible for the charge transfer/recombination processes.^1^ If two accumulation sites are available, corresponding to *N* = 2, there are two possible transfer paths, as schematized in Figure 2. Using eq 5, we can thus simulate IMPS spectra, employing *I*_hole_ = 1, *N* = 2, *k*_1_^tr^ = *k*_1_^rec^ = 1, *k*_2_^tr^ = *k*_2_^rec^ = 10 and *p_n_* ranging from 0 to 1. The resulting spectra together with the relative fit and DRT curve are reported in Figure 3.

From these calculations, we can extract some important features in IMPS spectra and GL-DRT curves. As expected, IMPS spectra are characterized by one negative semicircle at high frequency and a distorted positive semicircle at lower frequency. On the other hand, in GL-DRT curves, there is only one positive peak at low characteristic times (high frequencies), while more than one negative peak appears at higher characteristic times (low frequencies), except for the boundary cases with *p_n_* = 0 and *p_n_* = 1, where only one negative peak is displayed. The positive peak is centered exactly at *RC* = τ_cell_ = 10^–5^ s, meanwhile the other negative peaks are centered at characteristic time τ_1_^max^ = (*k*_1_^tr^ + *k*_1_^rec^)^−1^ = 0.5 s and τ_2_^max^ = (*k*_2_^tr^ + *k*_2_^rec^)^−1^ = 0.05 s.

As we saw in the previous paragraph, the peculiar feature of the L-DRT algorithm, is to return not only the right position of the peaks but also their height. In order to associate a physical meaning to the height of these peaks, it is necessary to rearrange eq 5 in a form of an admittance similar to eq S2, which allows the *g*(τ_*n*_) factor for every addendum to be identified. A detailed description of all the approximations necessary to this goal is reported in the second paragraph of the SI. After this operation, it turns out that the IMPS signal at steady state, i.e., when ω = 0, can be expressed in a very simple and convenient form as:
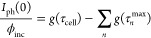
6where:
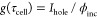
7a
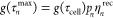
7bwith  representing the fraction of accumulated minority carriers that recombine with majority carriers at the surface of the semiconductor (recombination efficiency). Equation 7b suggest that the parameters *g*(τ) obtained with the L-DRT analysis have an important physical meaning when associated to the generalized RCM of a PEC system. In fact, the value *g*(τ_cell_) represents the fraction of holes accumulated at the semiconductor/electrolyte interface with respect to the total amount of incident photons which is, by definition, the product LHE × CSE. In Peter’s RCM, this quantity is the MFI. Instead, the value *g*(τ_*n*_^max^) represents the fraction of accumulated minority carriers at surface (in the *n*th site) that undergo recombination with majority carriers coming from the bulk with respect to the total amount of incident photons.

However, eq 7b shows that it is not possible to determine both *k*_*n*_^tr^ and *k*_*n*_^rec^ for each accumulation site, since *g*(τ_*n*_^max^) is proportional to the product *p_n_*η_*n*_^rec^. The following equation is obtained by putting together all the parameters extracted from L-DRT analysis, i.e., *g*(τ_cell_), *g*(τ_*n*_^max^), and τ_*n*_^max^, giving:
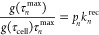
8

**Figure 4:**
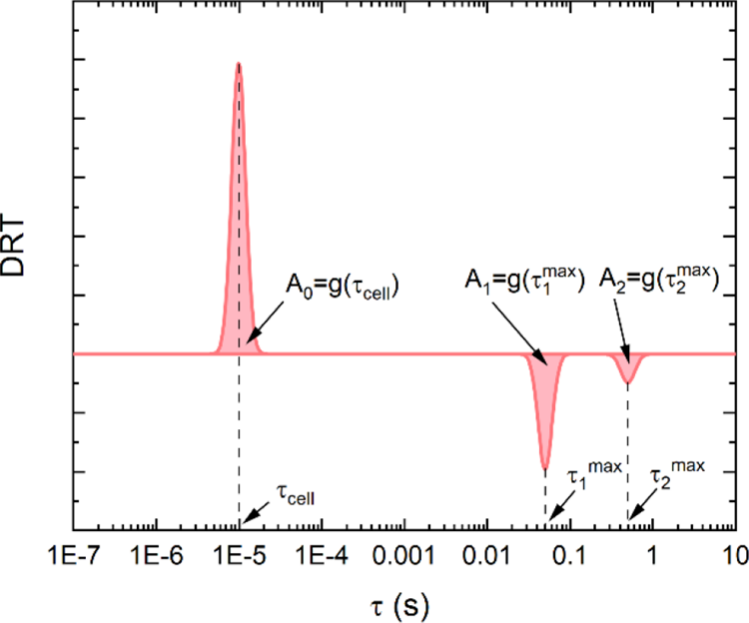
GL-DRT curve reported together with the main parameters coming from the L-DRT analysis. *A*_0_, *A*_1_, *A*_2_ are the integrals (area, highlighted in pink) of the respective Gaussian centered at τ_cell_, τ_1_^max^, and τ_2_^max^. Notice that the height of the Gaussian peak is normalized so that its integral is equal to the value *g*(τ_*n*_).

This relation highlights that the height of the peak of a GL-DRT curve cannot be directly correlated only to the kinetic rate constant *k*_*n*_^rec^ and *k*_*n*_^tr^ without knowing the fraction of holes *p_n_* that follow each path. If the various *p_n_* can be estimated by complementary time-resolved spectroscopic techniques,^25,26^ then a full quantitative analysis of each peak in the DRT spectrum can be achieved. Nevertheless, even without knowing *p_n_*, the L-DRT approach based on the generalized RCM provides a more comprehensive description of the charge dynamics at the semiconductor/electrolyte interface, with respect to the simple Peter’s RCM and the graphical inspection of the shape of the IMPS curve in a Nyquist plot. Figure 4 summarizes the main parameters that are determined from the L-DRT analysis.

Indeed, the *g*(τ_*n*_) parameter obtained using the L-DRT algorithm can be readily used for calculating other important physical quantity, such as the Gärtner current *I*_Gartner_(27) and the recombination current *I*_rec_. In several works,^1,2,4,10^ it was pointed out that the photocurrent measured in the external circuit is given by the difference between the flux of holes toward the surface *I*_Gartner_ (given by the Gärtner equation) minus the recombination current of electrons *I*_rec_ with holes trapped at the surface. These two currents have opposite sign since both electrons and holes flow toward the surface:
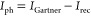
9

Comparing eq 9 with eqs 6 and 7b, it is possible to write that *I*_Gartner_ = ϕ_inc_*g*(τ_cell_) = *I*_hole_ and . L-DRT provides therefore an easy separation of the Gärtner current *I*_Gartner_ from the recombination current *I*_rec_.

Furthermore, L-DRT analysis enables an easy calculation of the rate constants *k*_LD_^tr^ and *k*_LD_^rec^, which describe the overall dynamic behavior at the semiconductor/electrolyte interface of the photoelectrochemical system. This can be achieved by approximating a system characterized by *N* negative peaks to a system with a single negative peak and transfer efficiency η_LD_^tr^. This peak will be characterized by a characteristic time τ_LD_^max^, given by the average of all the τ_*n*_^max^ relative to negative peaks weighted by their height *g*(τ_*n*_^max^):
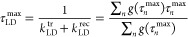
10

We can thus write the following equation:
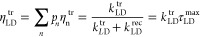
11

From eqs 10 and 11, it is now possible to calculate *k*_LD_^tr^ and *k*_LD_^rec^. The calculation of such parameters is then independent from the shape of the IMPS curve in the Nyquist plot, providing a more reliable analysis with respect to the graphical analysis used in Peter’s RCM.

### 2.3 Validation of L-DRT Analysis: Case Study of a Ti-Doped Hematite Photoanode

**Figure 5:**
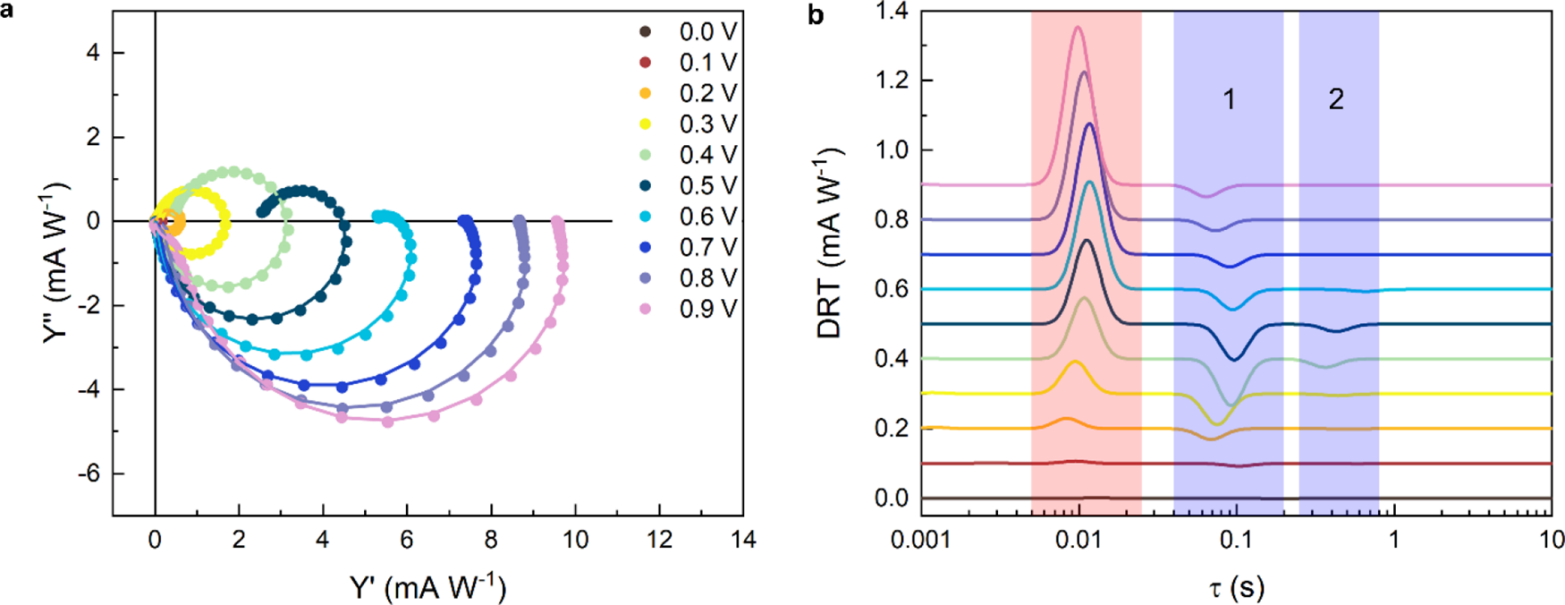
(a) IMPS spectra of the hematite photoanode together with their fir curve at different potential applied. (b) GL-DRT curves (λ = 0.5) of the IMPS spectra recorded at different applied potential. Measurements performed in borate buffer (0.25 M, pH 9.5) versus Ag/AgCl reference electrode.

In order to validate the L-DRT analysis, we applied it to a real system, namely a PEC cell for water splitting with Ti-doped hematite as a photoanode. The preparation procedure of the sample is reported in the SI. Hematite photoanodes represent a largely studied system and therefore they are suitable benchmarks for the present analysis. Linear sweep voltammetry (LSV) (Figure S1) and photoelectrochemical impedance spectroscopy (PEIS) (Figure S2) were performed in borate buffer aqueous solutions (0.25 M, pH 9.5) to characterize the photoelectrochemical properties of the photoanode. The voltammogram shows the typical delayed onset of the photocurrent due to the surface recombination, deviating from the ideal form predicted by the Gärtner equation.^27^ In fact, in hematite, charge recombination in the bulk and at the surface causes a decrease in the photocurrent, especially at low applied potential, when the band bending is still limited. We performed IMPS measurements at different applied potentials using the same illumination condition adopted for LSV. The amplitude of the sinusoidal signal of the incident light intensity was 10% (0.5 mW/cm^2^) of the continuous light bias (5 mW/cm^2^). Then we performed the fit of IMPS spectra using the L-DRT algorithm. Results are reported in Figure 5a together with the GL-DRT curves at different applied potentials (Figure 5b). As expected, here we observe a positive peak (highlighted in red) at around 0.01 s, whose height, expressed in A/W, is proportional to *g*(τ_cell_) and represents the fraction of photogenerated holes accumulated at the semiconductor/electrolyte interface with respect to the total amount of incident photons. The increasing height of this peak with the applied potential is strictly related to the increasing band bending at the surface of the electrode, which promotes charge separation and therefore charge collection at the surface. At around 0.1 s, there is a first negative peak (1, highlighted in blue), whose height is proportional to *g*(τ_1_^max^) and is related to accumulated holes that undergo recombination. Its height depends on the applied potential as well and shows a maximum at around 0.4 V_Ag/AgCl_. It is interesting to notice that the value of *C*_trap_ obtained from PEIS (see Figure S2a in the SI) has a maximum at around the same potential (slightly higher than 0.4 V_Ag/AgCl_), suggesting a strong correlation between these quantities. We believe that this correlation deserves further investigations and could provide deeper insight into the relation between PEIS and IMPS; however, this is beyond the aim of this study. Finally, the rise of a second recombination peak (2, highlighted in blue) at around 0.3–0.4 s in the interval 0.3 and 0.6 V_Ag/AgCl_ can be noticed, whereas the corresponding DRT spectra are hardly displaying any distortion, proving how the proposed analytical method allows for a deeper understanding into the charge dynamics. This weaker peak, impossible to discern with conventional IMPS analysis, may be related to the population of an additional set of surface states, ostensibly long-lived deep hole traps, resulting in a second recombination pathway characterized by the longest characteristic time. In perspective, the ability to resolve such additional peaks is even more important when dealing with complex systems, such as heterojunctions or photoelectrodes with functional layers, where potential-dependent characteristic relaxation times may be observed due to interfacial transfer processes.

**Figure 6:**
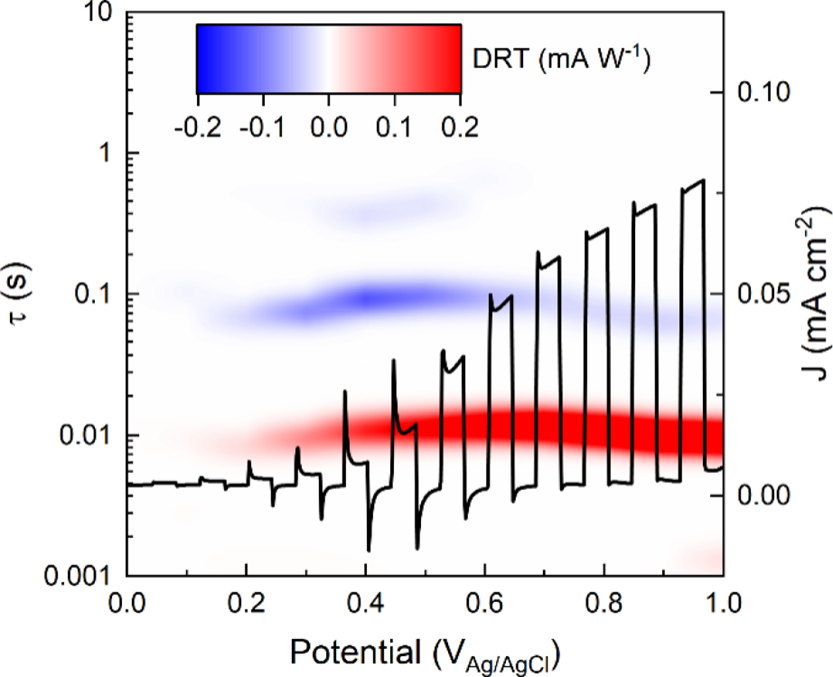
G L-DRT map obtained by putting together data from Figure 5b, overlapped with the relative chopped linear sweep voltammetry. Measurements performed in borate buffer (0.25 M, pH 9.5) versus Ag/AgCl reference electrode.

In order to highlight the dependence of IMPS-derived relaxation times on the potential applied to the PEC cell, we introduce the use of a novel 2D color map, as reported in Figure 6. Here, the horizontal and the left vertical axes are the applied potential and the time scale, respectively; the height of the GL-DRT curve (vertical scale in Figure 5b) is now represented in false colors, using red and blue tones for the positive and negative peaks of the DRT, respectively. Furthermore, the relevant (chopped) LSV can be reported on the same map (current density values on the right vertical axis), making it easier to correlate the specific characteristic time of each recombination process to the photocurrent characteristics.

**Figure 7:**
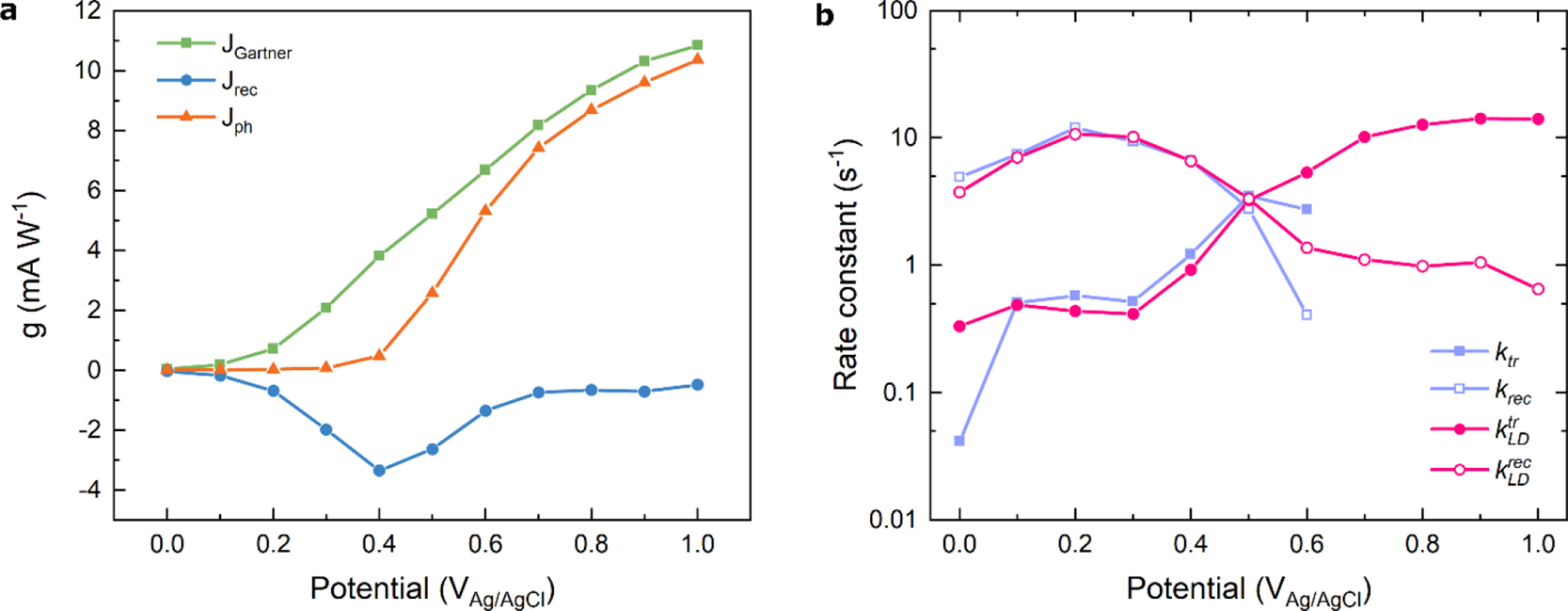
(a) Chopped linear sweep voltammetry of the hematite photoanode overlapped with *I*_Gartner_, *I*_ph_, and *I*_rec_. (b) Comparison between the rate constant *k*^tr^ and *k*^rec^ calculated using the RCM (blue) and *k*_LD_^tr^ and *k*_LD_^rec^obtained using the L-DRT algorithm (purple).

The positive signal at 0.01 s and the negative signal at 0.1 s start at the same potential, where the onset of a transient photocurrent appears also in the chopped LSV. The transient photocurrent in the chopped LSV exhibits a strong overshoot in the applied potential window between 0.3 and 0.6 V_Ag/AgCl_, suggesting strong electron–hole recombination. As already mentioned, in the same potential windows, the negative DRT signal at 0.1 s shows a maximum and the second negative signal at 0.3–0.4 s appears. The GL-DRT map turns out to be very effective in showing at first sight the dependence of recombination processes on the applied potential and how this modifies their dynamics. At this point, eqs 6, 7b, and 9 can be used to calculate *I*_Gartner_, *I*_rec_, and *I*_ph_. The resulting currents, normalized by the incident power of the incoming light, are reported in Figure 7a. The straightforward calculation of these quantities provided by the use of the L-DRT algorithm, even in high potential range, where usually the contribution of *I*_rec_ is difficult to evaluate since its value is close to zero, provides a powerful tool for the performance diagnostic of PEC systems in several experimental conditions, such as different wavelength or incident light intensity, composition of electrolyte, or even the structure of the photoelectrode, such as the doping concentration, the surface morphology, or the catalyst deposited on top of the surface.

Finally, in Figure 7b we compare the rate constants *k*^rec^ and *k*^tr^ obtained using the RCM, i.e., through graphical inspection of the IMPS features (from the LFI and ω_max_), to those determined analytically through the L-DRT analysis (see eqs 10 and 11).

The comparison shows that at low applied potentials (<0.6 V vs V_Ag/AgCl_) the two methods yield rather similar values of the rate constants. However, since no first-quadrant semicircle appears in IMPS above 0.6 V_Ag/AgCl_, it is impossible to extract *k*^rec^ and *k*^tr^ at higher potentials using the standard approach. In contrast, thanks to the L-DRT approach, it is possible to extract *k*_LD_^tr^ and *k*_LD_^rec^ over the full measurement range, since a negative DRT signal persists up to 1.0 V_Ag/AgCl_. The sigmoidal dependence of both *k*_LD_^tr^ and *k*_LD_^rec^ on the applied potential (*k*_LD_^rec^ > *k*_LD_^tr^ at low applied potentials and *k*_LD_^rec^ < *k*_LD_^tr^ at higher potentials) is typical of α-Fe_2_O_3_ and it has been widely investigated elsewhere.^6,28^

## 3 Conclusions

This paper presents a novel approach for the analysis of IMPS data, based on the calculation of the DRT curve using Lasso regression instead of the standard ridge regression. The adoption of Lasso regression is capable of removing the typical oscillations that affect the DRT curve obtained by using ridge regression when analyzing response functions with a negative imaginary part. As a consequence, the proposed algorithm greatly improves the ability of revealing hidden characteristic times of the system, providing a better time resolution and the correct intensity of processes related to them. This peculiar feature turns out to be particularly useful for analyzing IMPS data of semiconductors-based systems used for solar energy conversion, in particular, PEC systems. In fact, the features of a Gaussian L-DRT curve obtained from the application of the L-DRT algorithm on IMPS data of such systems, can be easily interpreted by adopting a generalized RCM. This approach allows to directly correlate the characteristic time τ_*n*_ of each peak in the DRT to the kinetic rate constants *k*_*n*_^rec^ and *k*_*n*_^tr^, and its height to the recombination efficiency η_*n*_^rec^. Further development of new physical models based on the results arising from the application of the proposed algorithm to IMPS data will be of great value in semiconductors photoelectrochemistry, as well as in photovoltaics, where the ability to resolve and distinguish the multiple charge transport processes is highly desired. In addition, the proposed method may be readily extended to complementary impedance spectroscopy techniques, such as PEIS and IMVS, providing a more realistic view of charge carrier dynamics in the μs to s range.

## Supporting Information Available

Description of the DRT-Lasso method, the physical approximations employed in analytical description of the model and additional experimental data on the PEC characterization of hematite photoanodes (PDF)
Python code developed for L-DRT analysis (PDF)
