# Supplementary material for: Distribution of Relaxation Times Based on Lasso Regression: A Tool for High-Resolution Analysis of IMPS Data in Photoelectrochemical Systems
Source: J Phys Chem C Nanomater Interfaces. 2023 Apr 20;127(17):7957–64. doi: 10.1021/acs.jpcc.3c00770 (PMC10166235; doi:10.1021/acs.jpcc.3c00770)
Supplement: Supplementary file 1 — jp3c00770_si_001.pdf [file jp3c00770_si_001.pdf]

Distribution of Relaxation Times based on Lasso regression: a tool for high resolution analysis of IMPS data in photoelectrochemical systems

Alberto Piccioni[a†], Pierpaolo Vecchi[a†], Lorenzo Vecchi[b], Silvia Grandi[c], Stefano Caramori[c], Raffaello Mazzaro[a*], Luca Pasquini[a].

[a] *Department of Physics and Astronomy, University of Bologna, Viale Berti Pichat 6/2, 40127, Bologna, Italy*
[b] *Department of Mathematics, University of Bologna, Piazza di Porta San Donato 5, 40126, Bologna, Italy*
[c] *Department of Chemical, Pharmaceutical and Agricultural Sciences, University of Ferrara, Via Luigi Borsari 46, 44121, Ferrara, Italy*
[†]*These authors contributed equally to this work*
[*]*Corresponding author:* raffaello.mazzaro@unibo.it

1. DRT-Lasso algorithm description

The DRT analysis models the admittance $Y(\omega)$ of a system with the integral equation

$$\int_0^\infty \frac{g(t)}{1+i\omega t}dt = Y(\omega) \in \mathbb{C} \qquad \text{S1}$$

where ω is the frequency at which the admittance $Y(\omega)$ is measured, $g(t)$ represents the unknown real-valued distribution over the times t that we want to find, and $i = \sqrt{-1}$ is the imaginary unit. From experimental measures, we only know $Y(\omega)$ for a finite number of frequencies $\{\omega_1, \dots, \omega_M\}$, such that the set $\left\{\tau_m = \frac{1}{2\pi\omega_m}\right\}_{m=1}^{M}$ is logarithmically evenly distributed in the range $[10^{-1}, 10^4]$. This problem is ill-posed in the sense of Hadamard[1], as the solution is certainly not unique (i.e. different distributions $g(t)$ give the same integral); therefore, a choice must be done on which kind of solution is preferred. Ideally, the resulting distribution should fit nicely the data $\{Y\ (\omega_1), \dots, Y\ (\omega_M)\}$ when plotted on the complex plane, without overfitting it.

From the physics of the system under investigation, it is reasonable to assume that the set of τ_m such that $g(\tau_m) \neq 0$ is discrete, i.e. there are very few characteristic times that fully describe the admittance of the system. The goal is to find this set of characteristic times with great precision. Once a characteristic time τ_m is found, the corresponding value $g(\tau_m)$ can be considered as its weight in describing the system. A positive (negative) weight means that the process related to that characteristic time increases (decreases) the photocurrent. Moreover, the weight can either be fully assigned to a single characteristic time using a Dirac distribution $g(\tau_m)\delta(\tau - \tau_m)$, or be distributed around τ_m using a Gaussian function with a physically reasonable standard deviation σ (see paragraph "Results and Discussion").

This problem will be tackled numerically with a discretization of the integral. The time range is subdivided in $N > M$ evenly logarithmically spaced intervals $[\tau_m, \tau_{m+1}]$, so that Eq. S1 becomes

$$Y(\omega_m) = \sum_{n=1}^{N} \frac{g(\tau_n)}{1 + i\omega_m \tau_n}, \qquad \text{S2}$$

for $n = 1, \dots, N$. Typically, $N = S \times M$ and a reasonable value is $S = 10$, meaning that the number of considered characteristic times is 10 times larger than the number the experimental frequencies. This results in a $(N \times M)$ complex linear system

$$Ag = b, \qquad \text{S3}$$

where $A \in \mathbb{C}^{M \times N}$ is the matrix $(A_{m,n}) = 1/(1 + i\omega_m \tau_n)$ and $b \in \mathbb{C}^M$ is the vector with the data $(Y(\omega_1), \dots, Y(\omega_M))$. An additional requirement for the vector $g \in \mathbb{R}^N$ is to be sparse, i.e. with 'many' zero-entries.

The linear system is first split it in real and imaginary part:

$$\begin{cases} A' g = b' \\ A'' g = b'' \end{cases} \qquad \text{S4}$$

where the two matrices A' and A'' have entries

$$(A'_{m,n}) = \frac{1}{1 + \omega_m^2 \tau_n^2} \qquad\qquad (A''_{m,n}) = -\frac{\omega_m \tau_n}{1 + \omega_m^2 \tau_n^2}$$

and b' and b'' are, respectively, the real and imaginary parts of b.

Then it is necessary to solve the following minimization problem

$$g = \arg\min_{x \in \mathbb{R}^N} \left(\left|\left|A'x - b'\right|\right|_2^2 + \left|\left|A''x - b''\right|\right|_2^2 + \lambda \left|\left|x\right|\right|_1 \right), \qquad \text{S5}$$

where $\| - \|_p$ denotes, as usual, the p-norm. In addition to the least square problem, the regularization term $\lambda \left|\left|x\right|\right|_1$ helps avoiding overfitting. Different choices can be made: in this work, we decide to introduce a Lasso regularization, as it is often used to achieve sparse solutions, penalizing solutions with a high number of non-zero entries[2].

The parameter $\lambda \in \mathbb{R}_{\geq 0}$ weights how much regularization is introduced in the minimization problem. Some validation tests have been developed[2] for identifying the best regularization parameter λ but these value may depend also on the quality of the measured data[3]. In general, if λ is too small, the regularization term becomes negligible and the solution is overfitted; if it is too big $(\lambda > 1)$, the regularization becomes prevalent, resulting in a solution which is

identically zero. In the following, we will always use $\lambda = 0.5$, since this value represents a reasonable trade-off for the present data analysis.

The algorithm was implemented in Python (main code attached as supporting material) and it is available here[4].

2. Physical approximations in the generalized RCM

In order to extract the height of the peaks that appears in the GL-DRT curves, it is necessary to rearrange Eq. 5 in a form of an admittance similar to Eq. S2, which allows the $g(\tau_n)$ factor for every addendum to be identified, as follows:

$$\frac{I_{ph}(\omega)}{\phi_{inc}} = \frac{I_{hole}}{\phi_{inc}}\left(\frac{1}{1+i\omega\tau_{cell}}\right)\sum_n p_n \frac{k_n^{tr}+i\omega}{k_n^{tr}+k_n^{rec}+i\omega}$$

$$= \frac{I_{hole}}{\phi_{inc}}\left(\frac{1}{1+i\omega\tau_{cell}}\right)\sum_n p_n \frac{k_n^{tr}-k_n^{rec}+k_n^{rec}+i\omega}{k_n^{tr}+k_n^{rec}+i\omega}$$

$$= \frac{I_{hole}}{\phi_{inc}}\left(\frac{1}{1+i\omega\tau_{cell}} - \frac{1}{1+i\omega\tau_{cell}}\sum_n \frac{p_n\eta_n^{rec}}{1+i\omega\tau_n^{max}}\right), \quad \text{S6}$$

where $\eta_n^{rec} = \frac{k_n^{rec}}{k_n^{tr}+k_n^{rec}}$ is the fraction of accumulated minority carriers that recombine with majority carriers coming from the bulk. Apart from the normalization factor I_{hole}/ϕ_{inc}, which is constant and does not depend on ω, Eq. S6 differs from Eq. S2 because of the factor $(1+i\omega\tau_{cell})^{-1}$ in front of the summation; however, this factor becomes appreciably different from 1 only at high frequencies, say $\omega\tau_{cell} \gtrsim 0.1$. In this range, the summation vanishes because $\tau_n^{max} \gg \tau_{cell}$, leading to $\omega\tau_n^{max} \gg 1$ and $(1+i\omega\tau_n^{max})^{-1} \approx 0$. This situation reflects the fact that the summation represents the low-frequency limit of the admittance, which is dictated by the long (compared to τ_{cell}) characteristic times τ_n^{max}. It is therefore legitimate to approximate the second, low-frequency term of Eq. S6 by setting $(1+i\omega\tau_{cell})^{-1} \approx 1$:

$$\frac{I_{ph}(\omega)}{\phi_{inc}} = \frac{I_{hole}}{\phi_{inc}}\left(\frac{1}{1+i\omega\tau_{cell}} - \sum_n \frac{p_n\eta_n^{rec}}{1+i\omega\tau_n^{max}}\right). \quad \text{S7}$$

In Eq. S7 it is useful to set $g(\tau_{cell}) = I_{hole}/\phi_{inc}$ and $g(\tau_n^{max}) = g(\tau_{cell})p_n\eta_n^{rec}$. If $k_n^{rec} \gg k_n^{tr}$ then $\eta_n^{rec} = 1$, meaning that all the minority carriers accumulated at the n-th sites recombines before going into the solution. On the other hand, if $k_n^{rec} \ll k_n^{tr}$ most of the minority carriers escape recombination and pass in the solution.

At steady-state, i.e. $\omega = 0$, Eq. S7 becomes:

$$\frac{I_{ph}(0)}{\phi_{inc}} = \frac{I_{hole}}{\phi_{inc}}\left(1 - \sum_n p_n \eta_n^{rec}\right) = \frac{I_{hole}}{\phi_{inc}}\left(1 - \sum_n p_n (1 - \eta_n^{tr})\right) = \frac{I_{hole}}{\phi_{inc}}\sum_n p_n \eta_n^{tr} = \frac{I_{hole}}{\phi_{inc}}\eta_{LD}^{tr} \quad \text{S8}$$

where $\eta_{LD}^{tr} = \sum_n p_n \eta_n^{tr}$ is the L-DRT transfer efficiency.

As expected, Eq. S8 suggests that the total steady-state photocurrent $I_{ph}(0)$ is given by the sum of the photocurrent that comes from every accumulation site. Eq. S8 can be also written by making explicit the efficiencies of the main optoelectronic processes involved in PEC systems, i.e. light harvesting efficiency (LHE), charge separation efficiency (CSE)[5], transfer efficiency η_{LD}^{tr} and external quantum efficiency (EQE). This results in:

$$\frac{I_{hole}}{\phi_{inc}}\sum_n p_n \eta_n^{tr} = LHE \times CSE \times \eta_{LD}^{tr} = EQE \quad \text{S10}$$

and therefore $\frac{I_{hole}}{\phi_{inc}} = g(\tau_{cell}) = LHE \times CSE$

3. Ti-doped hematite photoanode preparation

The photoanode fabrication and structural characterization was made by adapting and combining various previously reported synthetic approaches.[6,7] Ti(IV) doped nanostructured hematite photoanodes (Ti:Fe_2O_3) were prepared on a fluorine-doped SnO2 (FTO) conductive glass by an hydrothermal approach. Briefly, the FTO glass was cleaned through ultrasonication in isopropanol, and then rinsed with deionized water. The fabrication of hematite nano-rod electrodes involves the deposition of a Ti(IV) doped iron oxide seed layer by dip coating (0,625 mm/s) the cleaned 2 mm thick FTO/glass slides (10 mm (wide) X 25 mm (long) in a Fe(III) oleate precursor containing 15 mM Titanium (IV) isopropoxide in order to obtain a 10 mm X 10 mm coated area. The dip coating solution was prepared following the procedure described by D. K. Bora.[7] The Fe(III)oleate layer was converted into hematite following a 30 minute heat treatment at 500 °C. Solvothermal synthesis was carried out in a teflon-lined stainless steel autoclave by using an aqueous precursor containing 0,91 M sodium nitrate ($NaNO_3$, Carlo Erba Reagents), at a pH value of 1.5 adjusted with 6 M HCl, 0.136 M of ferric chloride ($FeCl_3$ · 6 H2O, Alfa Aesar), 2.5 mM Ti_2CN (Sigma-Aldrich) and a 5 % (v/v) ethanol (Carlo Erba Reagents). The seed-layered electrodes were inserted into the autoclave, lying with an angle of ca. 45° with respect to the vertical liner walls. Heating at 95 °C was applied for 4 h. A uniform layer of yellowish colour film (FeOOH) was formed on the electrodes. The FeOOH-coated substrates were washed with deionized water to remove weakly interacting residues from the hydrothermal batch before sintering in air at 550 °C for 1 h, during which conversion of FeOOH to Fe_2O_3 occurred. Finally, the resulting hematite thin films were modified by chemical bath treatment in a 0.2 M $TiCl_4$ solution heated at 50 °C for 1 hour, followed by a final thermal annealing at 760 °C for 10 minutes affording the Ti-Fe_2O_3 electrodes used for this study.

4. Photoelectrochemical characterization: Linear Sweep Voltammetry (LSV) and Photoelectrochemical Impedance Spectroscopy (PEIS)

LSV and PEIS were performed in order to have a solid basis as a starting point for our analysis. The illumination condition adopted for these measurements was the same used for IMPS measurements, namely 5 mW/cm^2 at 470 nm. LSVs were performed in dark, light and chopped illumination with a scan rate of 20 mV/s. Results are reported in Fig. S1.

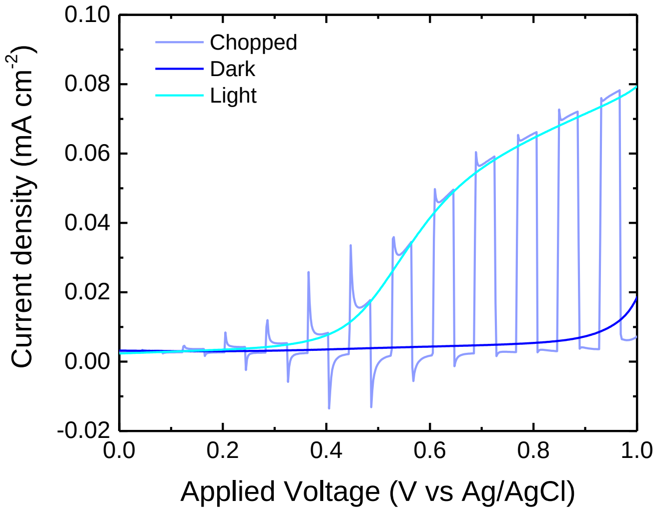

Figure S1: LSV performed with a blue (470 nm) LED at 5 mW/cm^2. Measurements performed in borate buffer (0.25M, pH 9.5) versus Ag/AgCl reference electrode.

The equivalent circuit used to fit the PEIS data is reported in Fig. S2a and was taken from the work by Bisquert et al.[8] The presence of a maximum in the capacitance C_{trap} (associated to trapping states on the surface) centered at around 0.5 - 0.6 $V_{Ag/AgCl}$ is a characteristic feature of hematite photoanodes and indicates the accumulation of charge carriers at surface states before the transfer to the electrolyte, that takes place at higher applied potentials. In order to validate the use of this circuit model, it is useful to compare the quantity dI/dV calculated from the LSV (Fig. S2b) in light with R_{tot}, the total resistance of the system, which in this case is given by

$$R_{tot} = R_S + R_{trapping} + R_{ct,trap}$$

where R_s, $R_{trapping}$ and $R_{ct,trap}$ were extracted directly from PEIS. These quantities are in good agreement, especially in the potential range where the photocurrent is different from zero, confirming the good choice of the circuit model.

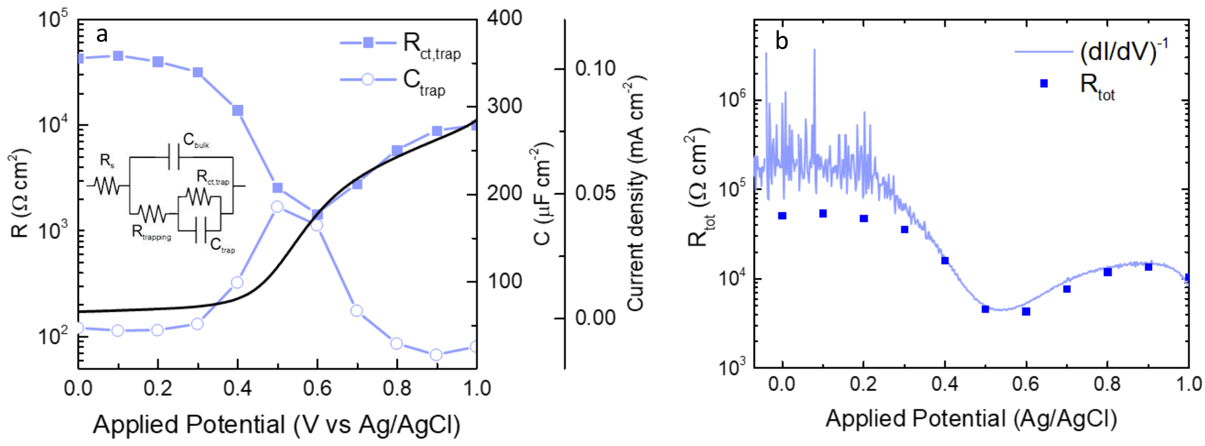

Figure S2: a) $R_{ct,trap}$ and C_{trap} calculated from PEIS measurements at different applied potentials; the circuit model is also reported. b) Comparison between $(dI/dV)^{-1}$ calculated from LSV in light and R_{tot} calculated from PEIS.

5. References

(1) Hadamard, J. Sur Les Problèmes Aux Dérivées Partielles et Leur Signification Physique. *Princet. Univ. Bull.* **1902**, *13*, 49–52.

(2) Saccoccio, M.; Wan, T. H.; Chen, C.; Ciucci, F. Optimal Regularization in Distribution of Relaxation Times Applied to Electrochemical Impedance Spectroscopy: Ridge and Lasso Regression Methods - A Theoretical and Experimental Study. *Electrochim. Acta* **2014**, *147*, 470–482.

(3) Hahn, M.; Schindler, S.; Triebs, L. C.; Danzer, M. A. Optimized Process Parameters for a Reproducible Distribution of Relaxation Times Analysis of Electrochemical Systems. *Batteries* **2019**, *5* (2).

(4) *https://github.com/nanolab-unibo/LassoDRT*.

(5) Rodriguez-Gutierrez, I.; Souza Junior, J. B.; Leite, E. R.; Vayssieres, L.; Souza, F. L. An Intensity Modulated Photocurrent Spectroscopy Study of the Role of Titanium in Thick Hematite Photoanodes. *Appl. Phys. Lett.* **2021**, *119* (7), 071602.

(6) Deng, J.; Zhong, J.; Pu, A.; Zhang, D.; Li, M.; Sun, X.; Lee, S. T. Ti-Doped Hematite Nanostructures for Solar Water Splitting with High Efficiency. *J. Appl. Phys.* **2012**, *112* (8).

(7) Bora, D. K. Fabrication of Silicon Doped Hematite Photoelectrode with Enhanced Photocurrent Density via Solution Processing of an In-Situ TEOS Modified Precursor. *Mater. Sci. Semicond. Process.* **2015**, *31*, 728–738.

(8) Klahr, B.; Gimenez, S.; Fabregat-Santiago, F.; Hamann, T.; Bisquert, J. Water Oxidation at Hematite Photoelectrodes: The Role of Surface States. *J. Am. Chem. Soc.* **2012**, *134* (9), 4294–4302.
