# Supplementary material for: Distribution of Relaxation Times Based on Lasso Regression: A Tool for High-Resolution Analysis of IMPS Data in Photoelectrochemical Systems
Source: J Phys Chem C Nanomater Interfaces. 2023 Apr 20;127(17):7957–64. doi: 10.1021/acs.jpcc.3c00770 (PMC10166235; doi:10.1021/acs.jpcc.3c00770)
Supplement: Supplementary file 2 — jp3c00770_si_002.pdf [file jp3c00770_si_002.pdf]

```
import numpy as np
import matplotlib.pyplot as plt
import cvxpy as cp
from scipy import interpolate
import numpy.matlib
from matplotlib.pyplot import cm
from google.colab import files
import pandas as pd

def cvxpy_solve_qp(A1, A2, b1, b2, tau, lambda_value):
    """
        this function conducts the quadratic programming with cvxpy and
        output the optimum in numpy array format
    """
    N_out = tau.shape[0]
    x = cp.Variable(shape = N_out, value = np.ones(N_out))
    h = np.zeros(len(tau))
    prob = cp.Problem(cp.Minimize(cp.norm(b1 - A1 @ x) + cp.norm(b2 - A2 @ x
) + lambda_value * cp.norm(x, 1)))

    prob.solve()
    gamma = x.value
    return gamma



def gaussian(tau, tau_c, A, sigma):
    return A*np.exp (-(np.log10(tau)-np.log10(tau_c))**2/(2*sigma**2))

# import data from txt file and construct tau array
# file needs to have frequency, real part and imaginary part in the first th
ree columns
filename = "" # insert file name
data = np.loadtxt(filename, skiprows = 0) # use skiprows to skip column name
s when importing data
f = data[:,0]
z_re = data[:, 1]
z_im = -data[:, 2]
f = f[::-1]
z_re = z_re[::-1]
z_im = z_im[::-1]
tau = 1/(2*np.pi*f)
# the considered characteristic times are logarithmically spaced and 10 time
s the experimental frequencies
tau = np.logspace(np.log10(tau[0]),np.log10(tau[-1]),10*len(tau))
# more tau values are added to expand the range of characteristic times used
 to fit the data
tau = np.insert(tau,0,tau[0:100]*10)
tau = np.append(tau,tau[-100:-1]/10)
N = len(f)
```

```
M = len(tau)

# define the matrices
A1 = np.zeros([N,M])
A2 = np.zeros([N,M])

for j in range(N):
  for k in range(M):
    A1[j,k] = (1/(1+omega[j]**2*tau[k]**2))
    A2[j,k] = (-(omega[j]*tau[k])/(1+omega[j]**2*tau[k]**2))

# carry out the Lasso regularization
g = cvxpy_solve_qp(A1, A2, z_re, z_im, tau, 1e-3)

# reconstructing the real and imaginary part using the values of g(tau)
z_re_fit = np.zeros(N)
z_im_fit = np.zeros(N)
for j in range(N):
  for k in range(len(tau)):
    z_re_fit[j] += (g[k]/(1 + 1j*omega[j]*(tau[k]))).real
    z_im_fit[j] += (g[k]/(1 + 1j*omega[j]*(tau[k]))).imag

# definition of the standard deviation used for the gaussian curves to build
 around each g(tau)
sigma = 10*(np.log10(tau[1])-np.log10(tau[0]))/np.sqrt(2*np.log(2))
g_gauss = np.zeros(len(tau))
g_g = np.zeros(len(tau))

for i in range(len(tau)):
  for j in range(len(tau)):
    g_g[j] = gaussian(tau[j], tau[i], g[i], sigma)
    g_gauss[j] += g_g[j]

# normalization of the height of each gaussian
area_peaks = np.trapz(y=g, x=np.log10(tau))
area_gauss = np.trapz(y=g_gauss, x=np.log10(tau))

g_gauss = g_gauss/area_gauss*area_peaks

# reconstructing the real and imaginary part using the values of g_gauss(tau
)
z_re_fit_gauss = np.zeros(N)
z_im_fit_gauss = np.zeros(N)
for j in range(N):
  for k in range(len(tau)):
    z_re_fit_gauss[j] += (g_gauss[k]/(1 + 1j*omega[j]*(tau[k]))).real
    z_im_fit_gauss[j] += (g_gauss[k]/(1 + 1j*omega[j]*(tau[k]))).imag

# plotting results of the fit and of the DRT data as peaks and gaussian
fig1,ax1=plt.subplots()
```

```
fig2,ax2=plt.subplots()
fig3,ax3=plt.subplots()

ax1.plot(z_re,z_im,'o',z_re_fit,z_im_fit,z_re_fit_gauss,z_im_fit_gauss,'--
',c=c)

ax2.semilogx(tau,g,c=c)

ax3.semilogx(tau, g_gauss,c=c)

# writing results on new files
fileFIT = open("/content/FIT.txt", "w")

for i in range(len(z_re_fit)):
  print(z_re_fit[i], '\t', z_im_fit[i], file=fileFIT)

fileFIT.close()

fileDRT = open("/content/DRT.txt", "w")

for i in range(len(tau)):
  print(tau[i], '\t', g[i], file=fileDRT)

fileDRT.close()

fileFITGAUSS = open("/content/FIT_gauss.txt", "w")

for i in range(len(z_re_fit)):
  print(z_re_fit_gauss[i], '\t', z_im_fit_gauss[i], file=fileFITGAUSS)

fileFITGAUSS.close()

fileDRTGAUSS = open("/content/DRT_gauss.txt", "w")

for i in range(len(tau)):
  print(tau[i], '\t', g_gauss[i], file=fileDRTGAUSS)

fileDRTGAUSS.close()

# download files
files.download("/content/FIT.txt")
files.download("/content/DRT.txt")
files.download("/content/FIT_gauss.txt")
files.download("/content/DRT_gauss.txt")
```
